# Liver transplantation in unresectable intrahepatic cholangiocarcinoma following neoadjuvant chemotherapy and SIRT

**DOI:** 10.1016/j.jhepr.2026.101830

**Published:** 2026-03-18

**Authors:** Baptiste Giguet, Florent Artru, Heithem Jeddou, Pauline Houssel-Debry, Marc-Antoine Jegonday, Valentin Coirier, Caroline Jezequel, Alexandre Chebaro, Fabien Robin, Karim Boudjema, Yan Rolland, Etienne Garin, Luc Beuzit, Bruno Turlin, Edouard Bardou-Jacquet, Julien Edeline, Thomas Uguen

**Affiliations:** 1 Department of Liver Disease, University of Rennes, CHU Rennes, Rennes, France; 2 Rennes Liver Failure Group (RELIEF), Rennes, France; 3 NuMeCan Institute INSERM UMR 124, Rennes, France; 4 Department of Hepatobiliary and Digestive Surgery, University of Rennes, CHU Rennes, Rennes, France; 5 Department of Radiology, University of Rennes, CHU Rennes, Rennes, France; 6 Department of Infectious Diseases and Medical Intensive Care, University of Rennes, CHU Rennes, Rennes, France; 7 Department of Medical Oncology, INSERM, University of Rennes, CLCC Eugène Marquis, COSS [(Chemistry Oncogenesis Stress Signaling)] – UMR_S 1242, Rennes, France; 8 Department of Pathology, University of Rennes, CHU Rennes, Rennes, France

**Keywords:** Liver Transplantation, Intrahepatic Cholangiocarcinoma, Selective internal radiation therapy

## Abstract

**Background & Aims:**

Unresectable intrahepatic cholangiocarcinoma (iCCA) has a poor prognosis, with limited curative options. Chemotherapy combined with selective internal radiation therapy (SIRT) has shown promising results in tumor response and survival. We evaluated liver transplantation (LT) outcomes following this neoadjuvant approach in patients with liver-limited iCCA in our center.

**Method:**

We retrospectively included all patients who underwent LT in the Rennes University Hospital for unresectable, locally advanced iCCA following neoadjuvant treatment with a combination of chemotherapy and SIRT.

**Results:**

Six patients underwent transplantation between 2010 and 2024. The median timeframe from diagnosis to listing was 351 days (IQR 250–558 days). The median time from listing to LT was 114 days (44–192 days). Age was 50.7 years (39.9–59.3 years) with a sex ratio of 1:1. The initial total tumor size was 100 mm (65–115 mm), with one tumoral lesion (1–1). Neoadjuvant treatment consisted of a gemcitabine–cisplatin regimen in four of six patients (66.7%), while one patient received cisplatin combined with 5-fluorouracil. The median follow-up was 4.9 years (1.8–8.8 years). The median length of hospitalization following LT was 12 days (10–20 days). Five-year overall survival was 100%, while 5-year progression-free survival was 44.4% (95% CI 8.9–88.0%) with three patients who experienced iCCA recurrence on Days 573, 577, and 1,180 after LT. No patient has yet died from tumor progression.

**Conclusions:**

This study presents one of the first series of unresectable iCCA cases treated with a neoadjuvant combination of SIRT and chemotherapy. Very selected patients (six patients over 15 years) who underwent LT demonstrated high long-term survival rates and an acceptable recurrence rate, even in the presence of large tumor sizes. Prospective trials evaluating this neoadjuvant combination are awaited.

**Impact and implications:**

Liver transplantation is not currently considered a standard therapeutic option for patients with unresectable, liver-limited intrahepatic cholangiocarcinoma because of historically poor outcomes. This study provides a scientific rationale for reconsidering transplantation in a highly selected subgroup of patients who achieve sustained disease control after neoadjuvant chemotherapy combined with selective internal radiation therapy. The results are particularly relevant for transplant hepatologists, oncologists, and surgeons, as they suggest that treatment response and prolonged disease stability may better predict post-transplant outcomes than tumor size alone. Clinically, these findings support a multidisciplinary, response-based selection strategy with a ‘test-of-time’ approach, while acknowledging the small sample size and retrospective design, and highlight the need for prospective studies and structured allocation frameworks to safely expand this approach.

## Highlights

• Neoadjuvant SIRT combined with chemotherapy was used for patients with unresectable, liver-limited iCCA.
• Selected patients undergoing liver transplantation achieved 100% 5-year overall survival, with good post-transplant recovery.
• Five-year progression-free survival was 44%, with manageable recurrence rates.
• Liver transplantation was feasible in ∼5.4% of the entire cohort (8/147) restricted to those with stable, liver-only, unresectable disease.

## Graphical abstract

**Figure:**
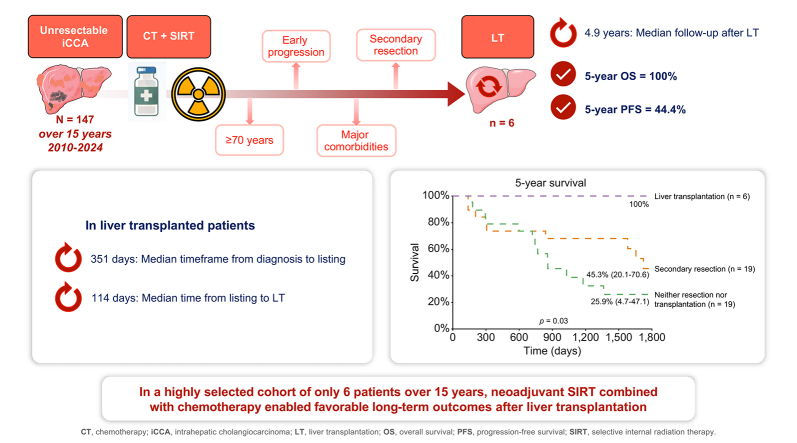

## Introduction

Cholangiocarcinoma (CCA), a malignancy originating from the biliary epithelium, includes distal, perihilar, and intrahepatic subtypes. Although rare, cholangiocarcinoma is the second most common primary liver cancer worldwide, with a rising incidence.^1^^,^^2^ Complete surgical resection with regional lymphadenectomy is the only curative treatment. However, only 10–15% of CCAs are resectable at diagnosis^3^ and even after complete R0 resection, outcome remains poor, with a 5-year survival rate of only 25%, primarily because of high rates of tumor recurrence.^4^ For unresectable CCA, standard treatment is systemic therapy and 5-year survival is <10%.^3^^,^^5^

For unresectable liver-limited peri-hilar CCA, neoadjuvant radio-chemotherapy followed by liver transplantation (LT) showed acceptable results in term of progression-free survival (PFS) and overall survival (OS).^6^ For unresectable intrahepatic CCA (iCCA), however, LT experiences achieved poor results in historical series with overall and recurrence-free survival rates of only 18–25% at 5 years [7,8]. As a result, unresectable iCCA not considered an indication for LT by international guidelines and in most expert centers.

Until recently, LT for iCCA was confined to incidental or misdiagnosed tumors identified post-transplantation, with limited neoadjuvant therapy utilization. Retrospective analyses have suggested that LT could be a better option than resection for early-stage iCCA^12^ and that patients with large unresectable iCCA selected by neoadjuvant treatment seemed to be good candidates for LT.^8^

Selective internal radiation therapy (SIRT), also known as yttrium-90 transarterial radioembolization, is a loco-regional treatment for liver malignancies. Radiolabeled microspheres are administered via the hepatic arteries, delivering radiation when reaching tumor vascular environment. A phase II study associating chemotherapy with SIRT for locally advanced iCCA demonstrated interesting results in survival and tumor response, suggesting that chemotherapy + SIRT could be an option as a bridge to surgery or even LT.^9^

Recently, Maspero *et al.*^10^ presented a limited experience of four patients who underwent LT for liver-limited unresectable iCCA after downstaging with systemic chemotherapy and SIRT with 100% PFS and OS after a median 26 months post-LT follow-up.

Since 2010, patients with unresectable liver-only iCCA are treated with combined chemotherapy and SIRT in our center.^13^ We also have shown in a phase II trial high response rates of 39%, with 22% of patients whose tumors were downsized and could be offered surgery, with interesting postoperative survival outcomes.^9^^,^^14^ Thus, leveraging extensive experience in SIRT for primary liver tumor lesions, we aimed to investigate the results of LT in patients with locally advanced unresectable iCCA with neoadjuvant treatment combining chemotherapy and SIRT.

## Patients and methods

We retrospectively included all patients who underwent LT in the Rennes University Hospital for unresectable, locally advanced iCCA following neoadjuvant treatment with a combination of chemotherapy and SIRT. Our center serves as the referral hub for these patients, and all cases are systematically reviewed within structured multidisciplinary team (MDT) meetings involving oncologists, surgeons, radiologists, and transplant hepatologists. Between 2010 and 2018, LT was considered on a compassionate basis if the aforementioned criteria were met. Since 2018 and the publication by Lunsford *et al.*,^15^ screening has been systematically performed during MDT meetings.

Criteria for undergoing LT were the following:

• Age below 70 years;
• Histologically proven iCCA with mass-forming measurable disease;
• Unresectable disease because of tumor location or severity of underlying liver disease, assessed by an experienced hepato-biliary surgical team;
• Absence of lymphatic, extrahepatic tumoral spread, or macrovascular invasion;
• Absence of uncontrolled disease (see below);
• Absence of contraindication to LT (cardiovascular, respiratory, oncological, performance status, addiction profile, etc.).

Exclusion criteria were:

• Mmultifocal iCCA with diffuse liver involvement;
• Concomitant malignancies or history of malignancy in the previous 5 years.

### Neoadjuvant therapy

Enrolled patients underwent chemotherapy (mostly with cisplatin 25 mg/m^2^ and gemcitabine 1,000 mg/m^2^ [reduced to 300 mg/m^2^ for the cycles just before and following SIRT]), administered intravenously on Day 1 and Day 8 of a 21-day cycle, with concomitant administration of SIRT with yttrium-90 glass microspheres (Therasphere, Boston Scientific, Marlborough, MA, USA) during cycle 2 or 3.

The SIRT procedure was performed as previously described.^15^ Percentage of pulmonary shunting and absence of digestive intake were assessed after 99mTc macroaggregated albumin was injected (185 MBq) during a first angiography. Planar and single-photon emission computed tomography (SPECT)-CT acquisitions were performed. SIRT was performed 8–15 days later at a second angiography, using glass microspheres.

All patients underwent restaging with computed tomography (CT)-scan and magnetic resonance imaging (MRI) every 2–3 months, before and after listing for LT. Fluorodeoxyglucose-positron emission tomography (FDG-PET) was performed during pre-LT checkup. Response to treatment was evaluated with CT scan according to Response Evaluation Criteria in Solid Tumors (RECIST) criteria.^16^

Before listing, patients underwent laparoscopically surgical lymph node sampling at stations 8 (common hepatic artery) and 12 (hepatoduodenal ligament).^17^

Cases were discussed in multidisciplinary meetings involving oncologists, hepatologists, radiologists and surgeons where stage and unresectability were initially confirmed and patients were proposed for neoadjuvant treatment and evaluation for LT. Cases were discussed on monthly basis up to placement on the waiting list. Placement on waiting list was collegially decided in patients with controlled oncological disease defined by at least 6 months response or stability under treatment and disease still deemed unresectable despite response. Depending on the response observed, tolerance of chemotherapy and expected waiting time, patients could either have chemotherapy holidays or maintenance chemotherapy with gemcitabine plus or minus cisplatin. In case of extrahepatic disease progression, patients were removed from the liver transplantation program.

### Statistical analyses

Categorical variables were expressed as number (percentage), whereas numerical variables were expressed as median (IQR). OS and disease-free survival (DFS) were calculated using the Kaplan–Meier method, with censoring at death or last follow-up: OS and DFS were calculated from LT. All analyses were conducted using NCSS v2024 (NCSS, LLC, Kaysville, UT, USA).

## Results

**Fig. 1:**
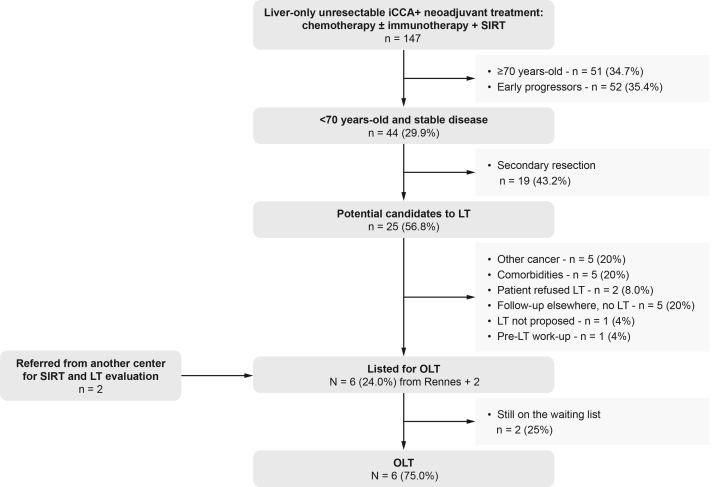
Flow-chart of the study.

**Fig. 2:**
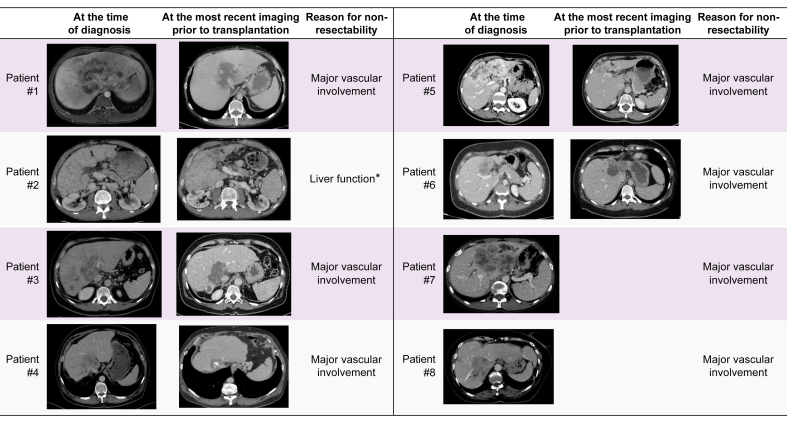
Illustration of non-resectability for each patient at the time of diagnosis and on the last imaging assessment performed within 3 months before liver transplantation. Patients 7 and 8 are still on the liver transplantation waiting list. Major vascular involvement was defined as bilateral or circumferential invasion of major vascular structures precluding satisfactory reconstruction (main portal vein or both right and left portal branches; inferior vena cava or major hepatic veins). **∗**Patient 2 had cirrhosis related to chronic Budd–Chiari syndrome. The cirrhosis was complicated by a histologically confirmed segment III cholangiocarcinoma, and non-resectability was related to the presence of clinically significant portal hypertension with a prior history of ascites, an episode of spontaneous bacterial peritonitis, and esophageal variceal rupture.

Between 1 January 2010 to 31 December 2023, 147 patients underwent SIRT for unresectable liver-limited iCCA in our center among which 51 (34.7%) were 70 years old or older. Non-resectability was attributable to tumor location and size, mainly with involvement of major vessels. Among patients who were <70 years old, 44 (45.8%) showed stable disease or response for longer than 6 months. Nineteen of them (43.2%) underwent secondary liver resection, while six of the 25 (24%) remaining were listed for LT. Two patients were specifically referred from another center for SIRT and LT evaluation, both were listed. Hence, a total of eight patients were listed and six (75%) were ultimately transplanted (Fig. 1). Illustrations of non-resectability for each patient at the time of diagnosis and on the last imaging assessment performed within 3 months before liver transplantation are provided in Fig. 2. The six LT accounted for 0.3% (6/1,783) of the total liver (only) transplants performed at our center during the study period. The median timeframe from iCCA diagnosis to listing was 351 days (IQR: 250–558 days), while the median time from listing to LT was 114 days (44–192 days).

The patients had a median age of 50.7 years (39.9–59.3 years) with a sex ratio of 1:1 between male and female. They exhibited a low level of comorbidities, as reflected by an age-adjusted Charlson Comorbidity Index of 3 (3–4). Two patients (33.0%) had liver cirrhosis, with a prior history of decompensation attributable to ascites. The median model for end-stage liver disease (MELD) score at the time of listing was 7 (7–11). The main characteristics of the patients are summarized in Table 1.

**Table 1:** Patients’ characteristics and liver transplantation variables.

	Population N = 6
**Patients’ characteristics and liver transplantation variables**	
Age, years	50.7 (39.9–59.3)
Sex, female	3 (50.0%)
Body mass index, kg/m^2^	23.2 (22.0–26.0)
Age-adjusted Charlson Comorbidity Index	3 (3–4)
MELD at listing	7 (7–11)
Diabetes	0 (0%)
Hypertension	1 (16.7%)
Tobacco consumption	2 (33.3%)
Cirrhosis	2 (33.3%)
Delay between diagnosis and listing, days	351 (250–558)
Delay between listing and liver transplantation, days	114 (44–192)
Donor risk index	1.91 (1.5–2.1)
Age of the donor, years	65 (55–88)
Sex of the donor, female	1 (16.7%)
Cold ischemia time, min	497 (403–512)

Categorical variables were expressed as number (percentage), while numerical variables as median (interquartile range). MELD, model for end-stage liver disease.

Regarding tumor characteristics, the initial total tumor size was 100 mm (65–115 mm), with a median of one tumoral lesion (1–1). At the time of listing, the total tumor size was 80 mm (52–80 mm). Neoadjuvant treatment consisted of the gemcitabine–cisplatin combination regimen (4/6, 66.7%), with cisplatin-5-fluorouracil used in one patient. One patient received treatment with durvalumab, an immune checkpoint inhibitor (ICI) in combination with gemcitabine–cisplatin. A 3-month interval was observed between the discontinuation of immunotherapy (durvalumab) and listing for LT. Lymph node sampling was performed in all but one patient, before waitlist placement in a median timeframe of 152 days (124–319 days), and results were negative in 100% (5/5) of cases (Table 3).

**Table 2:** Tumor treatments and characteristics.

	Population N = 6
**Tumor treatments and characteristics**	
Chemotherapy	
GEMCIS	4 (66.6%)
GEMCIS + DURVA	1 (16.7%)
LV5FU2 + CISPLATIN	1 (16.7%)
Selective internal radiation therapy (SIRT)	6 (100%)
Lymph node sampling	5 (83.3%)
Total tumor size at baseline	100 (65–115)
Number of tumor nodules at baseline	1 (1–1)
Total tumor size at listing	80 (52–80)
Number of tumor nodules at listing	1 (1–1)
Total tumor size on explant	70 (52–90)
Number of tumor nodules on explant	1 (1–4)
Tumor differentiation on explant	1 (1–2)
Lymphatic invasion on explant	0 (0%)
Perinervous invasion on explant	1 (16.7%)
Vascular invasion on explant	2 (33.3%)

Categorical variables were expressed as number (percentage), while numerical variables as median (interquartile range). DURVA, durvalumab; GEMCIS, gemcitabine + cisplatin; LV5FU2: 5-fluorouracil + calcium folinate. SIRT, selective internal radiation therapy.

**Table 3:** Outcomes following liver transplantation.

	Population N = 6
**Outcomes**	
Post-liver transplant hospitalization duration, days	12 (10–20)
Follow-up, years	4.9 (1.8–8.8)
Dindo–Clavien grade of complications	
1	1 (16.7%)
2	4 (66.7%)
3b	1 (16.7%)
Arterial complications	3 (50.0%)
Biliary complications	4 (66.7%)
Recurrence	3 (50%)
Time to recurrence, days	577 (573–33)
Sites of recurrence	
Hepatic	0
Extrahepatic	3 (100%)

Categorical variables were expressed as number (percentage), and numerical variables as median (interquartile range).

LT access was permitted in all patients in a specific situation in France known as ‘hors-tour’ which involves identifying donation after brain death, marginal grafts that have been declined by at least five teams as a result of donor-specific characteristics. Median donor age was 65 years (55–88 years), median cold ischemia time was 497 min (403–512 min), with a median donor risk index of 1.91 (1.5–2.1).

Liver pathology examination of explants revealed a median of one tumor (one to four), with a median tumor size of 70 mm (52–90 mm) with no lymphatic invasion while two (33.3%) had microvascular invasion and one (16.7%) perineural invasion. The individual histological analysis of the hepatic explants showed no complete tumor response (Table 2).

**Fig. 3:**
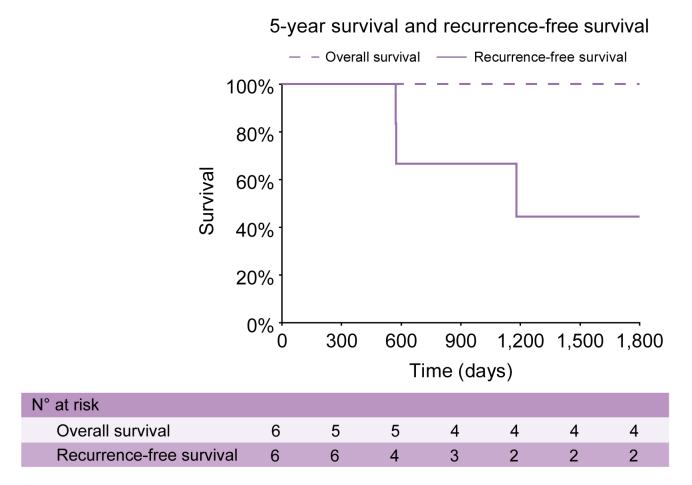
Five-year overall survival and recurrence-free survival among six patients transplanted in Rennes between 2010 and 2023 for liver-only, persistently unresectable intrahepatic cholangiocarcinoma after neoadjuvant therapy (chemotherapy ± immunotherapy 77; SIRT). Survival estimated by the Kaplan–Meier method. SIRT, selective internal radiation therapy.

Following LT, the median follow-up period was 4.9 years (1.8–8.8). Postoperative complications were categorized using the Dindo-Clavien classification: one patient (16.7%) experienced grade 1 complications, four patients (66.7%) had grade 2 complications, and one patient (16.7%) developed grade 3b complications (Table 3). Specifically, arterial complications occurred in three patients (50%) and biliary complications were observed in four patients (66.7%). Among them, three patients (75%) developed ischemic cholangitis with one (25%) requiring a retransplantation (after 12 years, with no evidence of recurrence on the explant), and one patient (25%) who died from recurrent cholangitis and septic shock (after 6.4 years). The last patient is still alive and undergoing endoscopic management for ischemic cholangitis. Additionally, one patient presented with a bilio-biliary anastomotic stricture. Notably, no cases of primary non-function were observed. The median length of hospitalization following LT was 12 days (10–20 days). Five-year OS was 100%, while 5-year PFS was 44.4% (95% CI 8.9–88.0%) (Fig. 3).

**Fig. 4:**
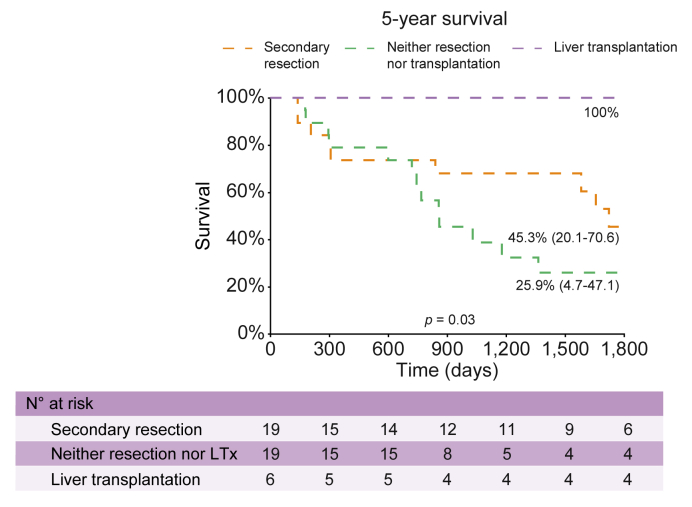
Five-year overall survival among six patients transplanted in Rennes between 2010 and 2023 for liver-only, persistently unresectable intrahepatic cholangiocarcinoma after neoadjuvant therapy (chemotherapy ± immunotherapy ± SIRT), compared with 19 patients who underwent secondary resection after neoadjuvant therapy and 19 patients who received neoadjuvant therapy but ultimately underwent neither resection nor transplantation. Survival estimated by the Kaplan–Meier method and compared with the log-rank test. Level of significance: *p* = 0.03.

We secondarily evaluated the 5-year OS of the three populations of interest after exclusion of non-transplantable patients based on age and early disease progression. Five-year OS of this cohort (n = 44) was 43.7 % (95% CI 27.7–59.8) (Fig. S1). When comparing outcome among the three populations, 5-year OS was 45.3% (95% CI 20.1–70.6%) in patients who underwent secondary resection (n = 19), 25.9% (95% CI 4.7–47.1%) in patients who were neither resected nor transplanted (n = 19), and 100% in patients who ultimately underwent LT (n = 6, *p* = 0.03) (Fig. 4).

Three patients experienced iCCA recurrence on days 573, 577, and 1,180 after LT. The patterns of recurrence were as follows: two cases (66.7%) involved pulmonary recurrence and were treated surgically with no novel recurrence so far, and one case (33.3%) presented as an isolated lymph node invasion and is treated with chemotherapy (gemcitabine + cisplatin) with a minor response at the last evaluation. Stereotactic body radiation therapy was recently completed for this patient. No patient has yet died from tumor progression.

All patients in the cohort received an immunosuppressive regimen including corticosteroids (intraoperative bolus followed by oral administration of 20 mg/day with gradual tapering and discontinuation between months 3 and 4 after LT), mycophenolate mofetil, and a calcineurin inhibitor, which was tacrolimus in 100% of cases. Everolimus was introduced in 66.7% of patients (4/6) between months 3 and 6 after LT, with discontinuation of mycophenolate mofetil and minimization of tacrolimus. Target trough levels were 3–8 ng/ml for everolimus and 3–5 ng/ml for tacrolimus. Two patients remain on long-term dual therapy with mycophenolate mofetil and tacrolimus.

## Discussion

In this single-center study of patients with locally advanced, unresectable iCCA treated with neoadjuvant therapy combined with chemotherapy and SIRT, followed by LT, we observed excellent long-term outcomes after a median follow-up of nearly 5 years, with a 5-year OS of 100% and a PFS of 44.4%. Notably, these outcomes were significantly higher compared with patients with liver-only unresectable iCCA treated with gemcitabine–cisplatin combined with SIRT, but without LT (respectively, 24% and 22%, but including 16 patients with subsequent resection after downsizing of the tumor).^18^ These outcomes were achieved in a very selected patients population (six patients over 15 years), in patients with large unifocal lesions who maintained disease control for approximately 1 year before being listed.

Historically, LT for unresectable iCCA showed very poor results in historical series (ranging from 30% to 42% at 5 years), and iCCA is not considered a valid indication for LT.^7^^,^^11^ However, recent advances in management and improved selection of candidates to LT have participated in increased OS and PFS chances following LT in recent series. Hence, in the study from McMillan *et al.*,^8^ of 18 patients who underwent LT for unresectable iCCA after neoadjuvant treatment, the OS at 5 years post-LT was 57%. Alongside with these results, a more recent series form Maspero *et al.*,^10^ reported good outcome following LT in four patients with locally advanced unresectable iCCA treated with a neoadjuvant treatment composed with chemotherapy and SIRT with 100% of patients being alive and free from disease recurrence at 73, 40, 12, and 8 months after LT.^10^ We reproduce this high level of survival rates in our series, with approximately 5 years median follow-up in our series.

The place of SIRT as a neoadjuvant option in patients with locally advanced iCCA is growing. Indeed, a recent prospective study has demonstrated promising results for the combination of SIRT and chemotherapy in locally advanced iCCA, achieving a response rate of 39%, which is superior to the 29% response rate observed with chemotherapy alone. This approach also achieved an OS of 21 months, with downstaging enabling secondary resection in 22% of patients.^9^ We also suggested that the results were improved over what is expected with chemotherapy only in liver-only iCCA [18]. Potential synergistic effects between chemotherapy and SIRT are not yet fully understood, although it is acknowledged that the radiation damage induced by SIRT may enhance the susceptibility of cancer cells to chemotherapy. The present findings could support the hypothesis that the neoadjuvant combination of SIRT and chemotherapy achieves effective disease control and aids in identifying optimal candidates for LT, characterized by favorable disease biology.

Selecting patients based on their response to neoadjuvant therapy seems critical in identifying optimal candidates for LT. Recently, Adam *et al.*^19^ demonstrated favorable outcomes for LT in cases of unresectable liver-only colorectal cancer using a comparable strategy, involving neoadjuvant therapy followed by transplantation, reserved for patients who achieved stable disease or showed a positive response to treatment. In our series, the median tumor size on explant was 7 cm, comparable with the findings of McMillan *et al.* (10 cm) and Maspero *et al.* (10 cm). This underscores the idea that, in this population, treatment response plays a significantly greater role than tumor size in predicting outcomes following LT. Moreover, we waited in median about 1 year after diagnosis to perform the LT, using a ‘test-of-time’ strategy to avoid transplantation of patients with rapid progression after first-line treatment. Sapisochin *et al.*^20^ reported that in patients undergoing LT, the presence of an undiagnosed iCCA with a tumor size >2 cm was associated with a poor prognosis. However, as those patients were untreated before LT, our results are not directly comparable to their findings and instead highlight the critical importance of achieving a favorable response to treatment before LT.

Despite this selection of patients with good results to treatment before LT, all patients had residual tumor in the explant. Albeit this can only be speculation, patients with residual tumor despite chemotherapy and SIRT would have been likely to progress locally during the 5-years median follow-up we achieved here. By contrast, in our six patients, all recurrences were oligo-metastatic progression that could be treated with local approaches. This suggest that LT had an important role in improving disease control, especially local control, and ultimately survival for these patients.

Importantly, the results of the present study convey an additional message. Although surgical resection is generally considered the gold standard once technically feasible, our findings suggest that this paradigm may deserve reconsideration and open further discussion regarding the respective roles of LT *vs*. secondary resection in this highly selected population. It is well established that only a minority of patients are candidates for curative-intent resection, with 60–80% being unresectable at diagnosis. Even after R0 resection, 5-year OS typically ranges between 25 and 40%, and recurrence rates often reach 50–80% within 2 years.[21], [22], [23] These data highlight that patients with solitary and technically resectable tumors constitute a biologically favorable subgroup compared with those presenting with multifocal, large, or vascularly invasive disease, as in our cohort. The present results appear superior to outcomes historically reported for resection in comparable tumor stages, particularly in patients who ultimately underwent LT, and to a lesser extent in those who underwent secondary resection.^23^ This likely reflects both the radicality of LT, enabling true R0 resection even in complex anatomical settings, and the stringent biological selection imposed by the requirement for sustained response or stability under neoadjuvant therapy.

Besides, Multifocal iCCA represents a biologically aggressive subtype and is consistently associated with significantly worse survival and higher recurrence rates after resection, as demonstrated across multiple large studies, where multifocality emerged as an independent adverse prognostic factor.^24^^,^^25^ Accordingly, multifocal disease was considered a strict contraindication in our selection algorithm, limiting the neoadjuvant–LT pathway to unifocal unresectable tumors, although future integration of biological markers such as circulating tumor DNA may allow identification of exceptionally favorable multifocal cases within controlled protocols.^26^

A key strength of our study is the consistency in pre-LT procedures and treatments, as 100% of patients received the upfront combination of chemotherapy and SIRT. In contrast, McMillan *et al.*^8^ reported that only 22% of patients received loco-regional therapy, primarily external beam radiation, and only as a consolidation treatment following disease stability under chemotherapy. Although our study population is smaller, the superior OS observed in our cohort may be attributed to the addition of SIRT to systemic therapy before LT. Unlike the approach described by McMillan *et al.*,^8^ we did not administer adjuvant therapy after LT. This decision was influenced by the unavailability of results from the BILCAP study during our study period and the previously negative outcomes of studies evaluating adjuvant therapy in resected CCA.^27^^,^^28^ Moreover, as our patients were treated with prolonged chemotherapy regimen before LT, the addition of capecitabine in the adjuvant setting was of uncertain benefit and might have been difficult in the post-LT context.

A limitation of our study is the small sample size, with only six patients undergoing LT for iCCA over a long study period questioning the selection process and the access to LT in potential candidates. Our center serves as the referral hub for these patients, and all cases are systematically reviewed within structured MDT meetings involving oncologists, surgeons, radiologists, and transplant hepatologists. This organization reduces—although does not eliminate—the risk of non-replicable clinical attitudes or treatment allocations. Finally, we observed that only one patient followed at our center who was potentially eligible for LT, with no apparent contraindications, was not identified as such and therefore did not undergo a pre-transplant work-up. The population was clearly heavily selected and cannot be proposed to the majority of patients with unresectable iCCA. However, the observed 5-year survival rate of 100% in this cohort is remarkable, especially when compared with the survival rates reported in the literature for initially unresectable iCCA treated with systemic therapy, even with recent immunotherapy-based regimen (36-month survival of 14.6% with cisplatin–gemcitabine–durvalumab in the TOPAZ-1 update, and of 13% with cisplatin–gemcitabine–pembrolizumab in the Keynote-966 update). This finding is thought-provoking and warrants further investigation. Besides, our study included mainly patients treated with chemotherapy without ICIs, which prevent us to draw any conclusions regarding the potential of processing LT in patients treated with recently adopted ICI-based regimen.^29^^,^^30^

In conclusion, this study presents one of the first series of unresectable iCCA cases treated with a neoadjuvant combination of SIRT and chemotherapy. Ultimately transplanted patients represented a highly selected subgroup (six patients over 15 years), accounting for 3% of the total cohort and 22% of potential transplantation candidates after exclusion of older patients, early progressors, and secondarily resectable cases. LT patients achieved high long-term survival and acceptable recurrence rates, even in the context of large tumor burdens. Prolonged response or stability under treatment appears to effectively identify good candidates for LT. Refining this approach could establish iCCA as a viable indication for LT. Prospective trials evaluating the neoadjuvant combination of SIRT and systemic therapy before LT for unresectable iCCA are essential to validate and expand these findings.

## Abbreviations

CCA, cholangiocarcinoma; CT, computed tomography; DFS, disease-free survival; FDG-PET, fluorodeoxyglucose-positron emission tomography; iCCA, intrahepatic cholangiocarcinoma; ICI, immune checkpoint inhibitor; LT, liver transplantation; MDT, multidisciplinary team; MELD, model for end-stage liver disease; MRI, magnetic resonance imaging; OS, overall survival; PFS, progression-free survival; RECIST, Response Evaluation Criteria in Solid Tumors; SIRT, selective internal radiation therapy; SPECT, single-photon emission computed tomography.

## Authors' contributions

Contributed to data collection, statistical analysis, medical record review, and manuscript writing: BG, FA, HJ, JE, and TU. Contributed to patient management and to the review and critical revision of the manuscript: PHD, MAJ, VC, CJ, AC, FR, KB, LB, BT, and EBJ.

## Data availability statement

Data will be made available upon reasonable request to the corresponding authors.

## Financial support

The authors did not receive any financial support to produce this manuscript.

## Conflicts of interest

BG reports receiving honoraria (speaker fees) and congress attendance funding from Gilead, Chiesi, and Astellas. FA reports receiving honoraria (speaker fees) from Gilead, AbbVie, Gore, Chiesi, Astellas, Mayoli, and Ipsen, as well as congress attendance funding from Gilead and AbbVie. YR reports receiving honoraria (speaker fees) from Boston Scientific. EG reports receiving consulting fees and honoraria (speaker fees) from Boston Scientific. JE reports contracts with BMS, BeiGene, Boston Scientific, Summit, AstraZeneca, Servier, Jazz, and Taiho; honoraria (speaker fees) from MSD, Eisai, BMS, AstraZeneca, Bayer, Roche, Ipsen, Basilea, Merck Serono, Incyte, Servier, BeiGene, Taiho, Boston Scientific, Guerbet, and Jazz; congress invitations from Roche and MSD; and participation in a monitoring board for Captor Therapeutics. HJ, PHD, MAJ, VC, CJ, AC, FR, KB, LB, BT, EBJ, and TU declare no conflicts of interest related to the subject.

Please refer to the accompanying ICMJE disclosure forms for further details.
