# Supplementary material for: Liver transplantation in unresectable intrahepatic cholangiocarcinoma following neoadjuvant chemotherapy and SIRT
Source: JHEP Rep. 2026 Mar 18;8(7):101830. doi: 10.1016/j.jhepr.2026.101830 (PMC13254992; doi:10.1016/j.jhepr.2026.101830)
Supplement: Multimedia component 1 [file mmc1.pdf]

Liver transplantation in unresectable intrahepatic cholangiocarcinoma following neoadjuvant chemotherapy and SIRT

B. Giguet, F. Artru, H. Jeddou, P. Houssel-Debry, M-A Jegonday, V. Coirier, C. Jezequel, A. Chebaro, F. Robin, K. Boudjema, Y. Rolland, E. Garin, L. Beuzit, B. Turlin, E. Bardou-Jacquet, J. Edeline, T. Uguen

Table of contents:

Fig. S1. 2

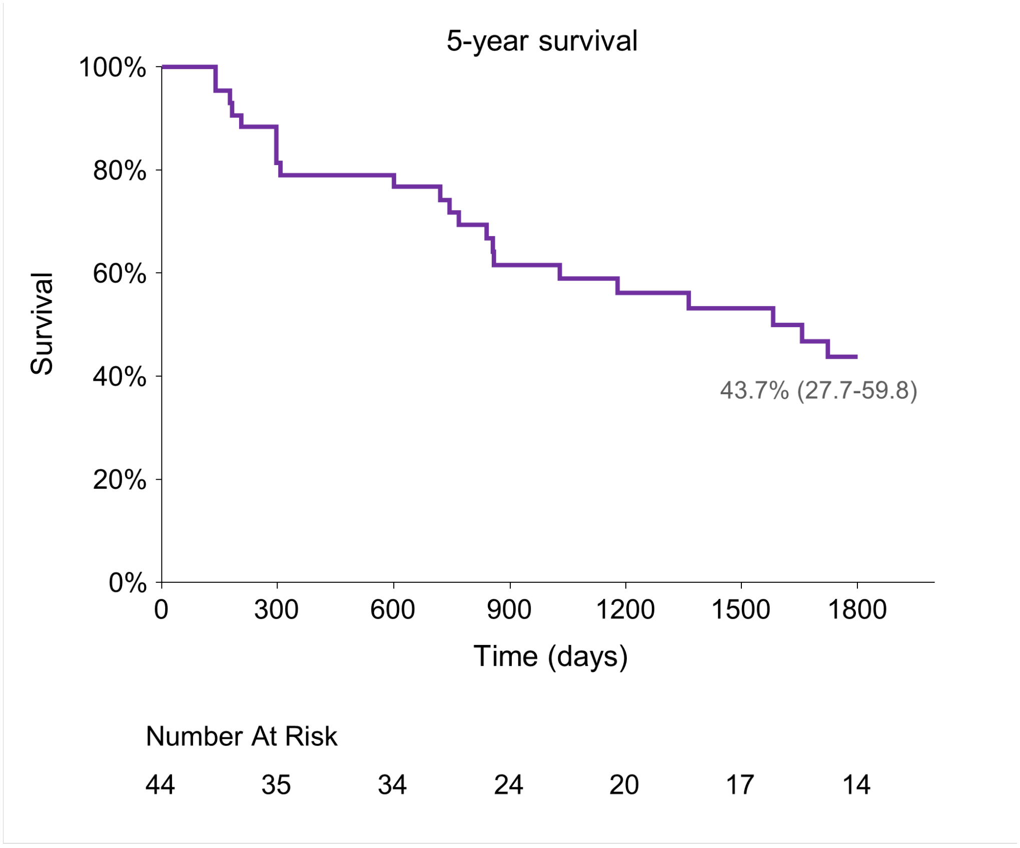

Fig. S1. Five-year survival among the 44 patients with liver-only unresectable iCCA, aged <70 years and with stable disease, treated with chemotherapy ± immunotherapy plus SIRT as neoadjuvant therapy.
